# BTOB: Extending the Biased GWAS to Bivariate GWAS

**DOI:** 10.3389/fgene.2021.654821

**Published:** 2021-05-06

**Authors:** Junxian Zhu, Qiao Fan, Wenying Deng, Yimeng Wang, Xiaobo Guo

**Affiliations:** ^1^Department of Statistical Science, School of Mathematics, Sun Yat-sen University, Guangzhou, China; ^2^Center for Quantitative Medicine, Duke-National University of Singapore Medical School, Singapore, Singapore; ^3^Department of Biostatistics, Harvard T.H. Chan School of Public Health, Harvard University, Boston, MA, United States

**Keywords:** GWAS, bivariate GWAS, summary association statistics, heritable covariate, biased

## Abstract

In recent years, a number of literatures published large-scale genome-wide association studies (GWASs) for human diseases or traits while adjusting for other heritable covariate. However, it is known that these GWASs are biased, which may lead to biased genetic estimates or even false positives. In this study, we provide a method called “BTOB” which extends the biased GWAS to bivariate GWAS by integrating the summary association statistics from the biased GWAS and the GWAS for the adjusted heritable covariate. We employ the proposed BTOB method to analyze the summary association statistics from the large scale meta-GWASs for waist-to-hip ratio (WHR) and body mass index (BMI), and show that the proposed approach can help identify more susceptible genes compared with the corresponding univariate GWASs. Theoretical results and simulations also confirm the validity and efficiency of the proposed BTOB method.

## 1. Introduction

Genome-wide association studies (GWASs) have been greatly successful in identifying tens of thousands susceptible genes for complex diseases or traits, revealing the genetic architectures of complex diseases or traits in question (Visscher et al., 2012, 2017). These large scale studies produce extremely valuable resource for further studies. However, due to the privacy concerns and other logistical considerations, most GWASs publish the summary association statistics rather than the individual-level data. This limitation motivates the rapid development of developing methods for analyzing the summary association statistics, such as conditional association analysis (Yang et al., 2012), gene-based association tests (Hu et al., 2013; Lee et al., 2013), jointly analyzing multiple traits (Zhu et al., 2015; Liu and Lin, 2018; Ray and Michael, 2018). A recent publication systematically reviews the development of summary association statistics-based methods (Pasaniuc and Price, 2017).

In this study, we mainly focus on the summary association statistics obtained from the GWASs of human diseases or traits while adjusting for heritable covariate, such as the GWAS of waist-to-hip ratio (WHR) after adjusting for BMI (Heid et al., 2010; Randall et al., 2013), the GWAS of fasting glycemic traits and insulin resistance after adjusting for BMI (Manning et al., 2012). However, it has been known that the results from these GWASs are biased, which may result in biased genetic estimates or even false positive genetic discoveries (Aschard et al., 2015). If the aim is to increase the statistical power, it is suggested to use the bivariate analyse of the trait (or disease) of interest and the corresponding heritable covariate (Aschard et al., 2015). However, the practical issue is still under addressed for this suggestion, that is how to extend the existing the biased GWAS to the bivariate analyse. Recent efforts have indicated that the multivariate GWAS can be conducted based on summary association statistics of the univariate GWASs (Zhu et al., 2015; Liu and Lin, 2018; Ray and Michael, 2018). However, these methods require the summary association statistics from the unbiased GWASs, that is the univariate GWASs without adjusting the heritable covariate. In reality, many studies only have the results from the GWAS after adjusting the heritable covariate. For example, in the GIANT (Genetic Investigation of ANthropometric Traits) consortium website, we can only download the summary association statistics for WHR adjusted BMI stratified by sex and age (Winkler et al., 2015). To obtain the results for WHR without adjusting for BMI, it needs to re-run a GWAS, which needs a great effort. To our best knowledge, there are no literatures addressing how to extend the biased GWAS to the bivariate GWAS.

In this paper, we develop a simple integration method called BTOB which extends the Biased GWAS TO Bivariate GWAS. We assess the valid and efficiency of BTOB using theoretical arguments and simulation studies. Finally, we apply the BTOB method to analyze the data downloaded from the GIANT consortium website.

## 2. Method

### 2.1. BTOB: Extending the Biased GWAS to Bivariate GWAS

Mathematically, the model used in the biased GWAS can be formulated as *Y*_2_ = *Gβ*_2_ + *Y*_1_γ_1_ + *Z*_2_ς_2_ + ε_2_, where *Y*_2_ is the trait or disease of interest, *Y*_1_ is the adjusted heritable covariate, *G* is the genotype score, and *Z*_2_ is the adjusted non-heritable covariates. In reality, many studies also had conducted additional GWAS for *Y*_1_, that is *Y*_1_ = *Gβ*_1_ + *Z*_1_ς_1_ + ε_1_. For example, the GIANT consortium had conducted the GWASs for WHR while adjusting for BMI, and the GWASs of BMI (Winkler et al., 2015). In addition, it is common that partial sample overlap between these two GWASs. For example, the sample size of the GWAS for BMI in men cohort with age greater than 50 is about 90,000, while the corresponding GWAS for WHR after adjusting BMI only use a sub-sample with about 60,000 sample. And the two studies may use different covariates adjustment strategies. In conclusion, the above real scenarios can be formulated as follows

(1) $$\begin{array}{l} \left(\begin{array}{c} Y_{1}^{c}\\ Y_{1}^{u_{1}} \end{array}\right)=\left(\begin{array}{c} G^{c}\\ G^{u_{1}} \end{array}\right)\beta_{1}+Z_{1}\varsigma_{1}+\varepsilon_{1}, \end{array}$$

(2) $$\begin{array}{l} \left(\begin{array}{c} Y_{2}^{c}\\ Y_{2}^{u_{2}} \end{array}\right)=\left(\begin{array}{c} G^{c}\\ G^{u_{2}} \end{array}\right)\beta_{2}^{*}+\left(\begin{array}{c} Y_{1}^{c}\\ Y_{1}^{u_{2}} \end{array}\right)\gamma_{1}+Z_{2}\varsigma_{2}+\varepsilon_{2}. \end{array}$$

Where $Y_{1}^{c}$ and $Y_{2}^{c}$ are the overlap sample of two phenotypes with genotypes *G*^*c*^, $Y_{1}^{u_{1}}$ is the unique sample only used in first model with genotypes $G^{u_{1}}$, and $Y_{2}^{u_{2}}$ and $Y_{1}^{u_{2}}$ are the unique sample only used in second model with genotypes $G^{u_{2}}$. *Z*_1_ and *Z*_2_ includes the intercept and covariates, which may consider different covariates for different GWAS. In Supplementary Theorem 1, we show that the estimates of the genetic effects ${\hat{\beta }}_{1}$ and ${\hat{\beta }}_{2}^{*}$ are independent. Under the null hypothesis *H*_0_: none of *Y*_1_ and *Y*_1_ associates with *G*, we have $(\frac{{\hat{\beta }}_{1}}{se\left({\hat{\beta }}_{1}\right)}{)}^{2}\sim \chi_{1}^{2},(\frac{{\hat{\beta }}_{2}^{*}}{se\left({\hat{\beta }}_{2}^{*}\right)}{)}^{2}\sim \chi_{1}^{2}$. Therefore, we can simply integrate the summary association statistics in model (1) and (2), that is

(3) $$\begin{array}{l} (\frac{{\hat{\beta }}_{1}}{se\left({\hat{\beta }}_{1}\right)}{)}^{2}+(\frac{{\hat{\beta }}_{2}^{*}}{se\left({\hat{\beta }}_{2}^{*}\right)}{)}^{2}\sim \chi_{2}^{2} \end{array}$$

which is a test statistics about testing the null hypothesis *H*_0_: none of *Y*_1_ and *Y*_2_ associates with *G*. Hence the proposed procedure extends the biased GWAS to bivariate analyse, which is termed BTOB (extends the Biased GWAS to Bivariate GWAS).

### 2.2. Simulations

We simulate 1,000 replicates of correlated traits, the causal SNP *G* is generated with minor allele frequency of 0.3 assuming the Hardy Weinberg equilibrium. The traits are generated using a linear additive model

$$\begin{array}{l} Y_{k}=\beta_{k}G+\varepsilon_{k},k=1,\ldots ,K \end{array}$$

where ${\left(\varepsilon_{1},\ldots ,\varepsilon_{K}\right)}^{⊤}$ follows multivariate normal distribution with mean 0 and covariance matrix Σ. We set the sample size of *Y*_1_ to be 5,000, and then vary the sample size of *Y*_2_ to be 5,000, 4,000, and 3,000. We consider three scenarios:(1) The tested variant affects the bivariate traits in the same direction. The tested variant explains 0.5% of the variance of *Y*_1_ and 0 to 0.5% of the variance of *Y*_2_, or the tested variant explains 0.5% of the variance of *Y*_2_ and 0 to 0.5% of the variance of *Y*_1_. The correlation was set to be low (ρ = 0.4), moderate (ρ = 0.6), or high (ρ = 0.8), where ρ was the correlation coefficient between *Y*_1_ and *Y*_2_. (2) The tested variant affects one phenotype only. Specifically, we considered the following two scenarios: the tested variant explains 0.5% of the variance of *Y*_1_ and 0% of the variance of *Y*_2_, or the tested variant explains 0.5% of the variance of *Y*_2_ and 0% of the variance of *Y*_1_. The correlation coefficient between *Y*_1_ and *Y*_2_ is varied from −0.9 to 0.9. (3) The test variant affects the bivariate traits in the opposite directions. The tested variant explains 0.3% of the variance of *Y*_1_ and 0.4% of the variance of *Y*_2_ with the opposite directions, or the tested variant explains 0.4% of the variance of *Y*_1_ and 0.3% of the variance of *Y*_2_ with the opposite direction. The correlation between *Y*_1_ and *Y*_2_ is varied from 0 to 0.9.

### 2.3. Study Decription

We download the gender and age specific summary association statistics for WHR after adjustment for BMI, and the marginal summary association statistics of BMI by the GIANT consortium from website http://portals.broadinstitute.org/collaboration/giant/index.php/GIANT_consortium_data_files (Winkler et al., 2015). We integrated the summary association statistics from the following univariate GWASs stratified by age and gender: BMI~SNP, WHR~SNP+BMI, resulting in the bivariate analysis of WHR and BMI. The aim of this study is to assess whether the proposed BTOB approach can contribute novel gene compared with the corresponding univariate GWASs. Hence, the gene is considered to be novel if the lead SNP in (or 400 KB flanking) a gene is genome-wide significant in the bivariate analysis, whereas none of the lead SNPs in (or 400 KB flanking) this gene reach genome-wide significance in the corresponding univariate GWASs. As we can only assess the HapMap II allele frequencies instead of pooled allele frequencies across all cohorts, we only included SNPs with sample size greater than 30,000, for which the HapMap allele frequencies may be representative.

## 3. Result

### 3.1. The Performance of BTOB in Integrating the Summary Association Statistics

For illustrate purpose, we conducted simulation studies to investigate the validity and efficiency of the proposed BTOB. As a comparison, we include the MANOVA method (Ray et al., 2016). Since MANOVA is not directly applicable to the summary association data, we use the overlap sample and re-run the multivariate association analysis using the MANOVA.

**Figure 1:**
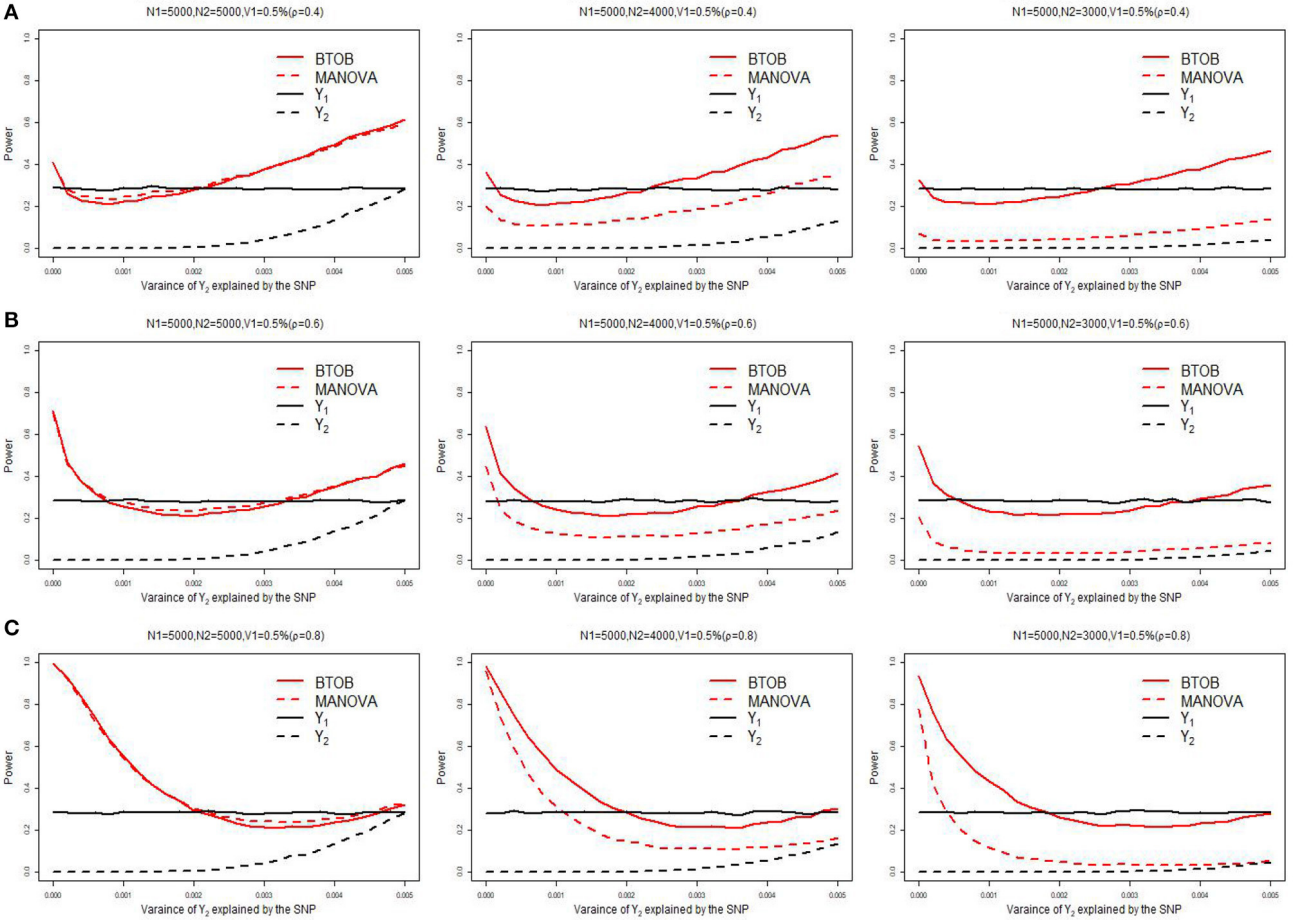
Power comparison of BTOB, MANOVA, and the univariate analysis. The test variant explains 0.5% of the variance of *Y*_1_, and the proportion of the test variant's variance for *Y*_2_ varies from 0 to 0.5%. The genetic effects of *Y*_1_ and *Y*_2_ are with the same direction. The sample size of *Y*_1_ is 5,000, and the sample size of *Y*_2_ is set to be 5,000, 4,000 and 3,000, respectively. Three levels of correlation between *Y*_1_ and *Y*_2_ are investigated: low correlation with ρ = 0.4 **(A)**, moderate correlation with ρ = 0.6 **(B)**, and high correlation with ρ = 0.8 **(C)**.

Supplementary Table 1 presents the type 1 error for BTOB, which shows that the proposed BTOB can control the type 1 error rate quite well. Figure 1 presents the power comparisons when the tested variant affects the bivariate phenotypes in the same direction. The tested variant explains 0.5% of the variance of *Y*_1_ and 0 to 0.5% of the variance of *Y*_2_. We can observe from Figure 1 that BTOB and MANOVA have nearly the same power when both phenotypes have the sample size 5,000, which indicates the validity and efficiency for BTOB. However, as the overlap sample size is set to be 4,000, BTOB performs much better than MANOVA. When the overlap sample size is set to be 3,000, the power discrepancy between BTOB and MANOVA is more obvious. This is expected as the sample size used in GWAS: *Y*_1_ = μ_1_ + β_1_*G*+*Z*_1_ς_1_ + ε_1_ is often larger than the sample size used in GWAS: *Y*_2_ = β_2_*G*+*Y*_1_γ_1_ + *Z*_2_ς_2_ + ε_2_. Traditional multivariate approaches, such as MANOVA, are only applicable to the overlap sample between *Y*_1_ and *Y*_2_. However, the proposed BTOB can make full use of the whole sample for *Y*_1_, hence boosting the power compared with MANOVA. The same phenomenons can be observed in Figures 1B,C with median and high correlation. In Figure 1, we also compare the power between the bivariate analysis and the univariate analysis after the Bonferroni correction. We can observe from Figure 1 that BTOB approach performs better than the univariate approach in most scenarios. It should be noted that there is a decrease of power for BTOB when the proportion of the test variant's variance for *Y*_2_ varies from 0 to a reasonably small value. This counterintuitive phenomenon can be explained by using the theoretical results given in a recent work (Guo et al., 2018). Supplementary Figures 1–3 present the power comparison for two other scenarios: the tested variant affects one trait only, and the tested variant affects the bivariate traits in the opposite direction. All of the simulated results indicate the superior performance for BTOB compared with MANOVA when the overlap sample size is set to be 4,000 and 3,000, and the superior power for BTOB compared with univariate analysis in most scenarios.

### 3.2. Real Data Analysis

In total, 8 loci are novel compared with the univariate GWASs: 4 for bivariate analysis of WHR and BMI in the cohort of men aged over 50, and 4 for bivariate analysis of WHR and BMI in the cohort of women aged over 50 (Table 1). The genomic control (GC) inflation factors of these 4 bivariate analyses is presented in Supplementary Table 2.

**Table 1:** The novel Genome-wide Significant loci which were identified by the proposed combining method but not found by the standard univariate approach for the analysis of WHR and BMI.

				**BMI**			**WHR~BMI**				**BTOB**	
**Cohort**	**SNP**	**Chr**	**Gene**	**Beta**	**SE**	***P-value*^a^**	***N*_1_**	**Beta**	**SE**	***P-value*^a^**	***N*_2_**	***P-value*^b^**
Men(Age>50)	rs10923746	1	WARS2	–0.020	0.0051	5.3e-05	90,515	0.029	0.0063	4.4e-06	56,398	5.405e-09
Men(Age>50)	rs12073056	1	TBX15	–0.022	0.0049	6.7e-06	90,142	0.030	0.0062	9.9e-07	55,682	1.774e-10
Men(Age>50)	rs3817973	6	HCG23	–0.018	0.005	2.7e-04	91,470	0.031	0.0062	4.7e-07	56,924	3.019e-09
Men(Age>50)	rs9378213	6	HLA-DRA	–0.022	0.0051	1.6e-05	89,222	0.03	0.0063	3.2e-06	56,647	1.264e-09
Women(Age>50)	rs12998590	2	SLC38A11	–0.022	0.0054	6.3e-05	88,374	0.031	0.0067	3.2e-06	57,158	4.702e-09
Women(Age>50)	rs253393	5	POC5	–0.026	0.0058	8.40E-06	88,423	-0.026	0.0072	0.00024	57,159	4.24E-08
Women(Age>50)	rs6971365	7	KLF14	–0.017	0.0052	0.0013	104,946	0.033	0.0062	1.00E-07	71,909	3.09E-09
Women(Age>50)	rs11191295	10	TMEM180	0.017	0.0049	4.1e-04	97,313	–0.027	0.0058	3.3e-06	66,010	2.898e-08

a *The results for univariate phenotypes approach. The genome-wide Significant level is set to be 2.5E-08 with the Bonferroni correction.*

b *The results for the BTOB approach. The genome-wide Significant level is set to be 5E-08. Chr, chromosome; N1, the sample size of GWAS for BMI; N2, the sample size of GWAS for WHR adjusting for BMI*.

Firstly, for the analyses of WHR and BMI in the cohort of women aged over 50, we identified 4 novel genes compared with the univariate GWASs (WARS2, leading SNP: rs10923746, *p*-value = 5.405E-09; TBX15, leading SNP: rs10923715, *p*-value = 4.88E-11; HCG23, leading SNP: rs3817973, *p*-value = 3.019e-09; HLA-DRA, leading SNP: rs9378213, *p*-value = 1.264e-09) (Table 1). Even though these 4 leading SNPs show evidence of association in the univariate analyses: GWAS for WHR after adjusting BMI and GWAS for BMI, these univariate analyses have no enough power to reach the genome-wide significance. What is more, for the analyse of WHR and BMI in the cohort of women aged over 50, BTOB method identified 4 novel loci compared with the univariate GWASs (SLC38A11, leading SNP: rs12998590, *p*-value = 4.702e-09; POC5, leading SNP: rs253393, *p*-value = 4.24E-08; KLF14, leading SNP: rs6971365, *p*-value = 3.09E-09; TMEM180, leading SNP: rs11191295, *p*-value = 2.898e-08) (Table 1). The real data analysis suggested that the BTOB method is capable to integrate moderate signals from the corresponding univariate analyses, hence leading to the identification of novel genetic signals compared with the univariate analyses. Further, six identified loci from the BTOB method, including TBX15, WARS2, POC5, KLF14, HLA-DRA, SLC38A11, were confirmed in the follow-up GWASs with at least ten times larger sample size (Pulit et al., 2019; Zhu et al., 2020), suggesting BTOB can help identify novel genes in the GWASs when the sample size is limited.

Finally, several studies have suggested a potential causal role of these identified genes in adipose development and function. For example, animal models have demonstrated that the important role of WARS2 in regulating brown adipose tissue function and consequently lipid and glucose metabolism, by regulating mitochondrial respiration, leading to the increased glucose oxidation in brown adipose tissues (Pravenec et al., 2017; Ejarque et al., 2019). TBX15 encodes a T-box transcription factor (TF) that has shown to be involved in various aspects of adipose development and maintenance, also to be associated with body fat distribution (Singh et al., 2005; Zhang et al., 2020). It has also been implicated the transcription factor KLF14, a member of the Krupple-like factor family (KLF), plays a key role in energy homeostasis by regulating lipid and glucose metabolism, and adipogenesis via promoting adipocyte differentiation (Chen et al., 2005; Birsoy et al., 2008).

## 4. Discussion

There are several concerns that should be noted about multivariate approaches in GWAS. First, the proposed bivariate method or other multivaraite methods for summary association statistics from univariate GWASs have been shown to help identify novel genes compared with univariate GWASs. While the multivariate approaches can also fails some genes identified in the univariate GWASs. Hence, the multivaraite GWASs should be considered as a valuable compensation rather substitution for univariate GWASs. Second, there is no single multivariate method that is uniformly most powerful in all scenarios. Hence, it is valuable to try several candidate methods in real case.

In summary, our proposed approach provides an efficient shortcut for extending the existing biased GWASs to the bivariate GWAS. Considering a great amount of large scale biased GWASs have been published (Hancock et al., 2010; Kaplan et al., 2011; Randall et al., 2013; Loth et al., 2014; Winkler et al., 2015; Pulit et al., 2019; Zhu et al., 2020), the proposed BTOB method is expected to be of great practical utility.

## Data Availability Statement

The original contributions presented in the study are included in the article/Supplementary Material, further inquiries can be directed to the corresponding author/s.

## Author Contributions

XG conceived the idea and conducted the simulation. JZ, WD, YW, and QF processed the data and conducted the real dataset experiments. XG and QF wrote the manuscript. XG, QF, JZ, and WD revised the manuscript. All authors read and approved the final manuscript.

## Conflict of Interest

The authors declare that the research was conducted in the absence of any commercial or financial relationships that could be construed as a potential conflict of interest.
