# Supplementary material for: BTOB: Extending the Biased GWAS to Bivariate GWAS
Source: Front Genet. 2021 May 6;12:654821. doi: 10.3389/fgene.2021.654821 (PMC8134661; doi:10.3389/fgene.2021.654821)
Supplement: Supplementary file 1 [file Data_Sheet_1.pdf]

BtoB: Extending the biased GWAS to bivariate GWAS

Junxian Zhu, Qiao Fan, Wenying Deng, Yimeng Wang, and Xiaobo Guo

Supplementary Information

Index

Figure S1. Power comparison of Y_1, Y_2, BtoB, MANOVA (Part 1).

Figure S2. Power comparison of Y_1 , Y_2, BtoB and MANOVA (Part 2).

Figure S3. Power comparison of Y_1 , Y_2, BtoB and MANOVA (Part 3).

Table S1. The type 1 error of BtoB for integrating the summary statistics under different simulation settings. The significant level is set to be 0.001.

Table S2. Genomic inflation factor (λ) for the univariate GWAS for BMI, BMI-adjusted GWAS for WHR and the proposed BTOB approach for the analysis of WHR and BMI.

Table S3. Genomic inflation factor (λ) for the univariate GWAS for Anthropometrics stratified by sex and the proposed BtoB approach.

Table S4. Comparisons of the number of genome-wide Significant loci from two approaches: univariate phenotypes and the proposed BtoB approaches for the analysis of WHR and BMI.

Table S5. Comparisons of the number of genome-wide Significant loci from two approaches: univariate phenotypes and the proposed BtoB approaches for the analysis of Anthropometrics stratified by sex.

Table S6.The novel Genome-wide Significant loci which were identified by the proposed combining method but not found by the standard univariate approach without adjusting heritable trait.

Theorem S1.

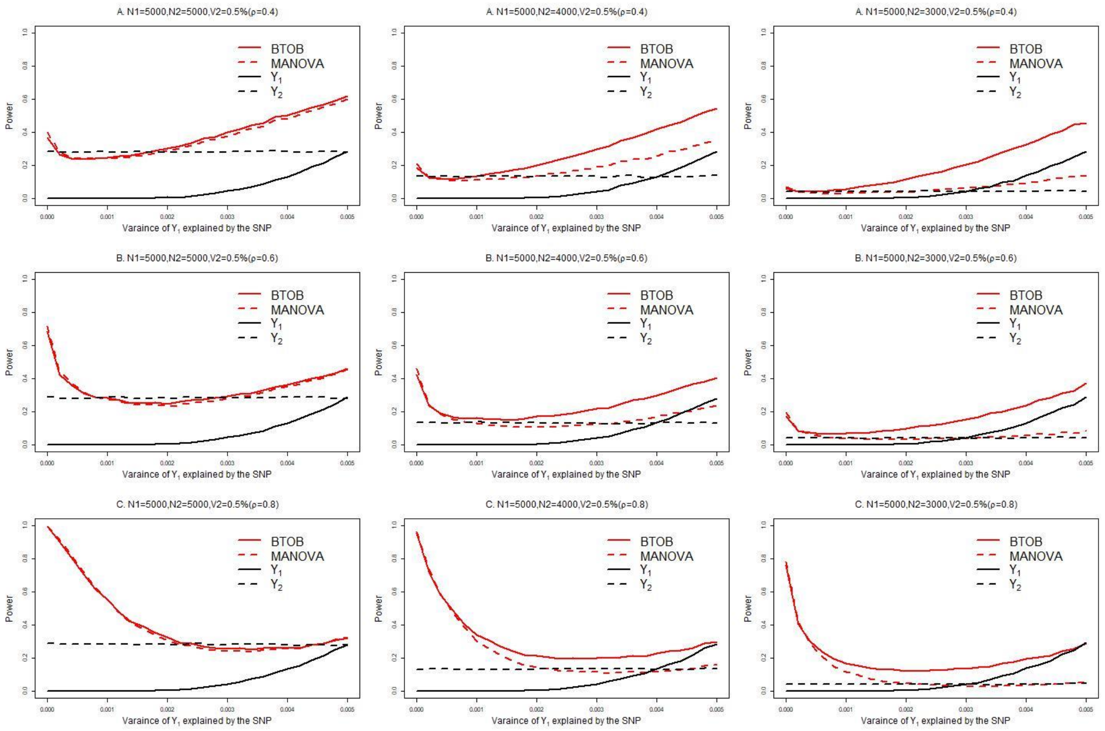

Figure S1. Power comparison of Y_1, Y_2, BtoB, MANOVA (Part 1). The tested variant explains 0.5% of the variance of Y_2, and the proportion of the tested variant's variance for Y_1 varies from 0 to 0.5%. The genetic effects of Y_1 and Y_2 are in the same direction. The sample size for Y_1 is 5,000, and the sample size of Y_2 is set to be 5,000, 4,000 and 3,000, respectively. Three levels of correlation between Y_1 and Y_2 are investigated: low correlation with ρ= 0.4 (A), moderate correlation with ρ= 0.6 (B) and high correlation with ρ= 0.8 (C).

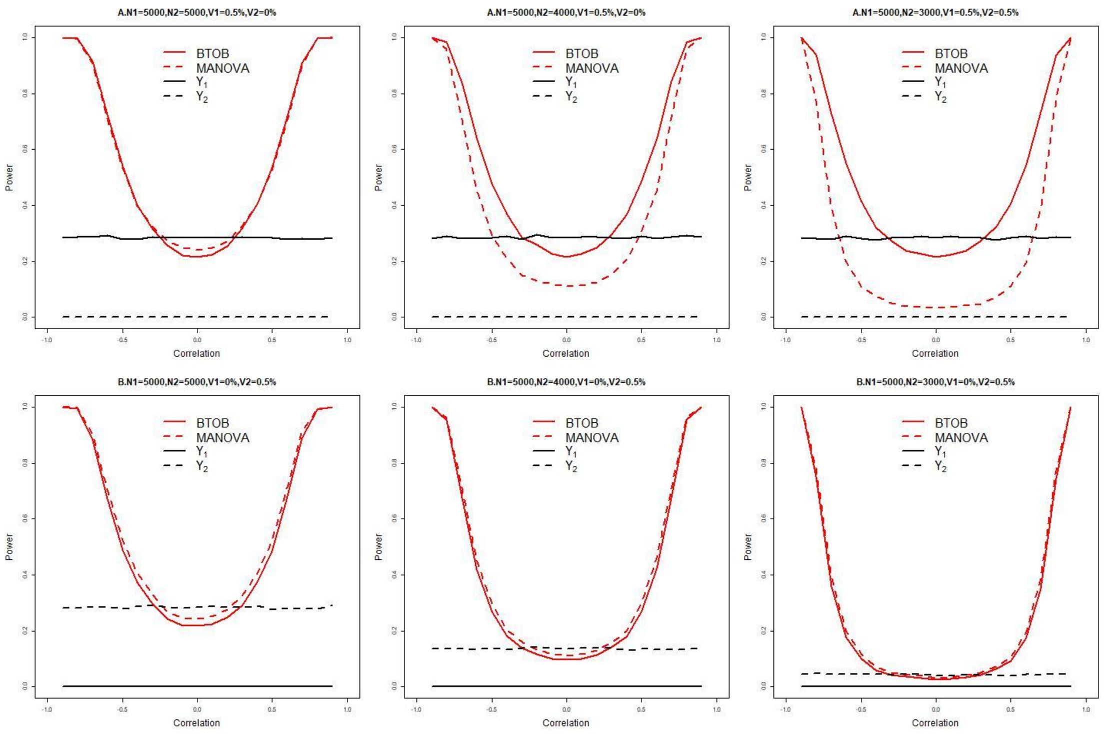

Figure S2. Power comparison of Y_1 , Y_2, BtoB and MANOVA (Part 2). (A) The tested variant explains 0.5% of the variance of Y_1 and 0% of the variance of Y_2, The sample size for Y_1 is 5,000, and the sample size of Y_2 is set to be 5,000, 4,000 and 3,000, respectively. The correlation between Y_1 and Y_2 varies from -0.9 to 0.9. (B) The tested variant explains 0.5% of the variance of Y_2 and 0% of the variance of Y_1. The sample size for Y_1 is 5,000, and the sample size of Y_2 is set to be 5,000, 4,000 and 3,000, respectively. The correlation between Y_1 and Y_2 varies from -0.9 to 0.9.

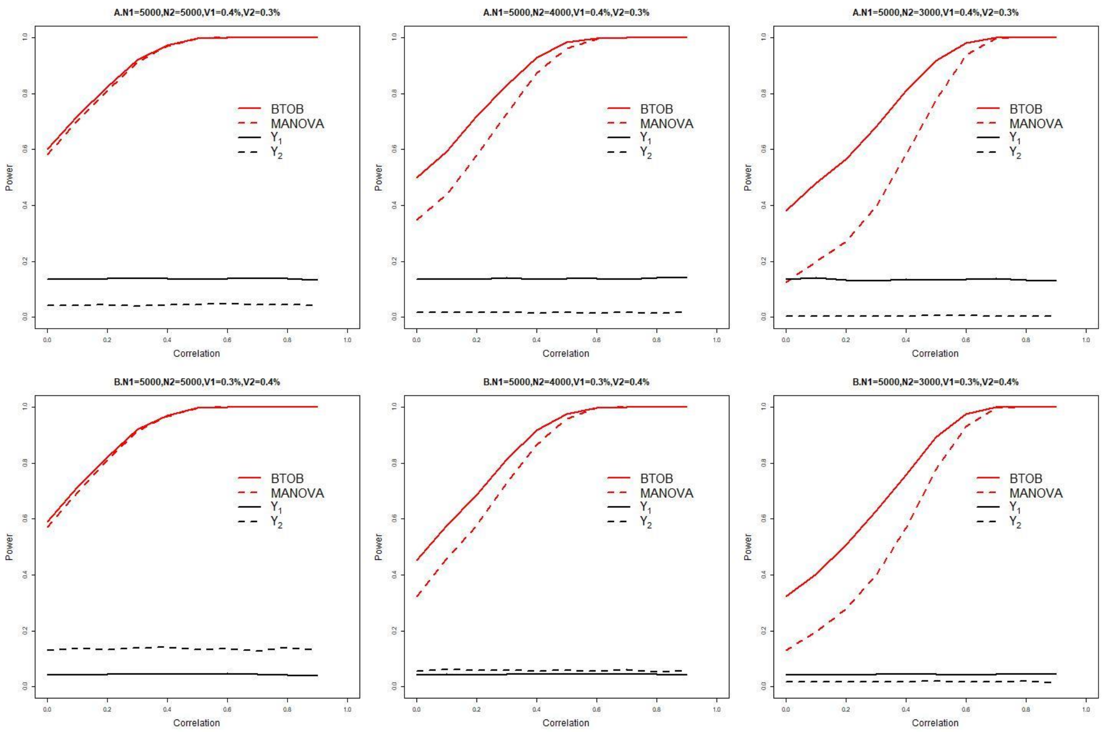

Figure S3. Power comparison of Y_1 , Y_2, BtoB and MANOVA (Part 3). (A) The tested variant explains 0.3% of the variance of Y_1 and 0.4% of the variance of Y_2 with the opposite direction. The sample size for Y_1 is 5,000, and the sample size of Y_2 is set to be 5,000, 4,000 and 3,000, respectively. The correlation between Y_1 and Y_2 varies from 0 to 0.9. (B) The tested variant explains 0.4% of the variance of Y_1 and 0.4% of the variance of Y_2, The sample size for Y_1 is 5,000, and the sample size of Y_2 is set to be 5,000, 4,000 and 3,000, respectively.

Table S1. The type 1 error of BtoB for integrating the summary statistics under different simulation settings. The significant level is set to be 0.001.

N_1	N_2	ρ	Type 1 Error
5000	5000	−0. 8	0. 001004
5000	5000	−0. 4	0. 000956
5000	5000	0	0. 001028
5000	5000	0. 4	0. 000932
5000	5000	0. 8	0. 001
5000	4000	−0. 8	0. 001036
5000	4000	−0. 4	0. 000998
5000	4000	0	0. 000924
5000	4000	0. 4	0. 001004
5000	4000	0. 8	0. 000982
5000	5000	−0. 8	0. 001004
5000	5000	−0. 4	0. 000956
5000	5000	0	0. 001028
5000	5000	0. 4	0. 000932
5000	5000	0. 8	0. 001

Abbreviations: N_1: the number of sample size for phenotype Y_1; N_2: the number of sample size for phenotype Y_2; ρ the correlation coefficient between phenotypes Y_1 and Y_2.

Table S2. Genomic inflation factor (λ) for the univariate GWAS for BMI, BMI-adjusted GWAS for WHR and the proposed BtoB approach for the analysis of WHR and BMI.

Cohort	BMI	WHR~BMI	BtoB
Men (Age>50)	1.03070	1.07456	1.01974
Men (Age<50)	1.05263	1.07456	1.04890
Women (Age>50)	1.00877	1.07456	1.01930
Women (Age<50)	1.03070	1.09649	1.03969

Theorem S1: The estimates $\widehat{\beta}_1$ and $\widehat{\beta}_2^*$ obtained in model (1) and (2) are independent.

Proof:

Let $Y_1 \overset{\text{def}}{=} \begin{pmatrix} Y_1^c \\ Y_1^{u_1} \end{pmatrix}$, $G_1 \overset{\text{def}}{=} \begin{pmatrix} G^c \\ G^{u_1} \end{pmatrix}$, $Y_2 \overset{\text{def}}{=} \begin{pmatrix} Y_2^c \\ Y_2^{u_2} \end{pmatrix}$, $Y_1^* \overset{\text{def}}{=} \begin{pmatrix} Y_1^c \\ Y_1^{u_2} \end{pmatrix}$, $G_2 \overset{\text{def}}{=} \begin{pmatrix} G^c \\ G^{u_2} \end{pmatrix}$. $e(Y_1|G_1, Z_1)$ denotes as the residuals for fitting the linear regression with response Y_1 and predictors G_1, Z_1, that is

$$e(Y_1|G_1, Z_1) \overset{\text{def}}{=} Y_1 - (G_1, Z_1)((G_1, Z_1)^T(G_1, Z_1))^{-1} \, (G_1, Z_1)^T Y_1.$$

Denotes $H_{G_1,Z_1} = (G_1, Z_1)((G_1, Z_1)^T(G_1, Z_1))^{-1} \, (G_1, Z_1)^T$, then $e(Y_1|G_1, Z_1) = \left(I - H_{G_1,Z_1}\right)Y_1$. Since $\widehat{\beta}_1$ and $\widehat{\beta}_2^*$ asymptotically follow the normal distribution, we only need to show $\text{cov}\left(\widehat{\beta}_1, \widehat{\beta}_2^* | G_1, G_2, Z_1, Z_2\right) = 0$. It can show that,

$$\widehat{\beta}_1 = \left(e(G_1|Z_1)^T e(G_1|Z_1)\right)^{-1} e(G_1|Z_1)^T e(Y_1|Z_1)$$

$$\widehat{\beta}_2^* = \left(e(G_2|Z_2, Y_1^*)^T e(G_2|Z_2, Y_1^*)\right)^{-1} e(G_2|Z_2, Y_1^*)^T e(Y_2|Z_2, Y_1^*).$$

Since $E\left(\widehat{\beta}_1|G_1, Z_1\right) = \beta_1$ and

$$E\left[\widehat{\beta}_2^*|G_2, Z_2\right] = E\left[E\left[\widehat{\beta}_2^*|G_2, Z_2, Y_1^*\right]|G_2, Z_2\right]$$

$$= E\left[\left(G_2{}^T(I - H_{Y_1^*,Z_2})G_2\right)^{-1} G_2{}^T(I - H_{Y_1^*,Z_2})E\left[(I - H_{Y_1^*,Z_2})Y_2\middle|G_2, Z_2, Y_1^*\right]|G_2, Z_2\right]$$

$$= E\left[\left(G_2{}^T(I - H_{Y_1^*,Z_2})G_2\right)^{-1} G_2{}^T(I - H_{Y_1^*,Z_2})E[Y_2|G_2, Z_2, Y_1^*]|G_2, Z_2\right]$$

$$= E\left[\left(G_2{}^T(I - H_{Y_1^*,Z_2})G_2\right)^{-1} G_2{}^T(I - H_{Y_1^*,Z_2})(G_2\beta_2^* + Y_1^*\gamma_1 + Z_2\varsigma_2)|G_2, Z_2\right]$$

$$= E\left[\left(G_2{}^T(I - H_{Y_1^*,Z_2})G_2\right)^{-1} G_2{}^T(I - H_{Y_1^*,Z_2})G_2\beta_2^*|G_2, Z_2\right]$$

$$= E[\beta_2^*|G, Z_1, Z_2] = \beta_2^*$$

we have

$$\text{cov}\left(\widehat{\beta}_1, \widehat{\beta}_2^*|G_1, G_2, Z_1, Z_2\right)$$

$$= E\left[\widehat{\beta}_1\widehat{\beta}_2^*\middle|G_1, G_2, Z_1, Z_2\right] - E\left[\widehat{\beta}_1\middle|G_1, Z_1\right]E\left[\widehat{\beta}_2^*\middle|G_2, Z_2\right]$$

$$= E\left[E\left[\widehat{\beta}_1\widehat{\beta}_2^*\middle|G_1, G_2, Z_1, Z_2, Y_1^*\right]\middle|G_1, G_2, Z_1, Z_2\right] - \beta_1\beta_2^*$$

$$= E\left[\widehat{\beta}_1 E\left[\widehat{\beta}_2^*\middle|G_2, Z_2, Y_1^*\right]\middle|G_1, G_2, Z_1, Z_2\right] - \beta_1\beta_2^*.$$

$$= E\left[\widehat{\beta}_1\beta_2^*\middle|G_1, G_2, Z_1, Z_2\right] - \beta_1\beta_2^* = 0$$

The proof is completed. ☐
